# The Regulatory Interplay of the Colorectal Cancer Biomarkers MACC1 and IER2 and Its Impact on Metastatic Cancer Survival

**DOI:** 10.3390/biom16030398

**Published:** 2026-03-07

**Authors:** Miguel Enrique Alberto Vilchez, Benedikt Kortüm, Paul Schöpe, Lenka Kyjacova, Fabian Zincke, Marc Osterland, Janice Smith, Wolfgang Walther, Beate Rau, Jonathan Paul Sleeman, Ulrike Stein

**Affiliations:** 1 Experimental and Clinical Research Center, Charité—Universitätsmedizin Berlin, and Max-Delbrück-Center for Molecular Medicine, Robert-Rössle-Straße 10, 13125 Berlin, Germany; miguel.alberto@charite.de (M.E.A.V.);; 2 Department of Surgery, Campus Charité Mitte and Campus Virchow-Klinikum, Charité—Universitätsmedizin Berlin (Corporate Member of Freie Universität Berlin, Humboldt-Universität zu Berlin, and Berlin Institute of Health), 13353 Berlin, Germany; 3 Medizinische Fakultät Mannheim, Universität Heidelberg, European Center for Angioscience (ECAS), Ludolf-Krehl-Str. 13–17, 68167 Mannheim, Germany; 4 Karlsruhe Institute for Technology (KIT), Institute for Biological and Chemical Systems-Biological Information Processing (IBCS-BIP), Hermann-von-Helmholtz-Platz 1, 76344 Eggenstein-Leopoldshafen, Germany; 5 German Cancer Consortium (DKTK), Neuenheimer Feld 280, 69120 Heidelberg, Germany

**Keywords:** MACC1, IER2, proliferation, metastasis, colorectal cancer

## Abstract

We have previously identified MACC1 and IER2 as functional biomarkers in the context of colorectal cancer. In silico correlation analysis suggested a possible functional connection between the expressions of these biomarkers, given that a significant positive correlation between IER2 and MACC1 RNA was observed. In loss- and gain-of-function experiments, we found that MACC1 positively regulates the expression of IER2. Furthermore, pulldown experiments provided evidence for MACC1-IER2 protein–protein interactions. Functionally, MACC1 enhanced proliferation of HCT116 cells overexpressing IER2 but not of HCT116 cells with knockdown of IER2 expression. Patients with high expressions of both biomarkers lived significantly shorter, whereas those with low concentrations of both markers showed the longest survival. Taken together, these findings show a functional interplay between the colorectal biomarkers MACC1 and IER2, which, in turn, has an impact on the survival of colorectal cancer patients.

## 1. Introduction

Colorectal cancer (CRC) is one of the most common cancer diagnoses worldwide and a leading cause of cancer-related morbidity and mortality. Furthermore, metastatic CRC is responsible for drastically reducing survival [1]. Personalization of treatments for CRC using patient molecular profiling technologies based on biomarker panels holds great promise for optimizing treatment efficacy and minimizing toxicity [2]. It is becoming increasingly evident that many of these biomarkers interact in ways that can impact prognosis and the efficacy of therapy [3]. Understanding these interactions offers the prospect of rational simultaneous targeting of interdependent pathways to enhance treatment efficacy, as well as sequential targeting, so that when one targeted therapy fails, alternative pathways can be inhibited. In addition, such knowledge should allow for the circumvention of pathway crosstalk that leads to resistance and the determination of the best order in which to apply targeted therapies.

In previous work we identified Metastasis Associated in Colon Cancer-1 (MACC1) as a key regulator of metastasis and tumor progression and a robust prognostic biomarker in more than 20 solid cancers, including CRC [4]. Meta-analyses [5,6] confirmed that MACC1 overexpression correlates with poor overall survival (OS) and disease-free survival (DFS) in solid tumors. In CRC, higher MACC1 expression in UICC stages I–III has been linked to an increased risk of metachronous metastasis, highlighting its prognostic significance.

In other studies, we found that expression of IER2, a member of the immediate early response gene family, correlates with poor metastasis-free and overall survival in patients with colorectal adenocarcinomas [7]. IER2 is rapidly induced by proliferation- and migration-inducing stimuli, such as p38 and JNK signaling, is strongly upregulated in a variety of cancers, and plays a functional role in metastasis [7]. At the molecular level, IER2 modulates the protein phosphatase 2 (PP2A) holoenzyme, affecting key targets such as HSF1 and CDC25A, which influence cell proliferation and apoptosis [8]. Additionally, IER2 has been linked to hepatocellular carcinoma (HCC) metastasis [9] and to poor prognosis in melanoma, where it promotes a senescence-associated secretory phenotype (SASP) via the p53/p21 axis [10]. These findings highlight IER2’s multifaceted role in cancer progression and its potential as a therapeutic target.

Here, we provide evidence for a cooperative interplay between MACC1 and IER2, in which MACC1 promotes IER2 expression, and IER2 promotes colony formation and sustained proliferation. This interplay results in a significantly worse prognosis for mCRC patients, as the coincident overexpression of these two genes identifies a patient subset with the shortest overall survival (OS).

## 2. Materials and Methods

### 2.1. Cell Culture

Cell lines SW480, SW620, HCT116, RKO, and DLD1, obtained from the American Type Culture Collection, were cultured in Dulbecco’s modified Eagle’s medium (DMEM) or Roswell Park Memorial Institute (RPMI) medium supplemented with 10% fetal bovine serum (FBS) (Thermo Fisher Scientific, Waltham, MA, USA). The SW480 cell lines with or without ectopic MACC1 expression (SW480/MACC1 and SW480/EV, respectively. EV: empty vector) were generated as previously described [11]. SW620 clones with short hairpin (shRNA) targeting MACC1 and their control counterparts (SW620/shMACC1 and SW620/shCtrl, respectively. shCtrl: short hairpin control) were also established according to previous protocols [11]. The HCT116/IER2 and HCT116/EV cell lines (with and without ectopic IER2 expression) were provided by Prof. Jonathan Sleeman from the University of Heidelberg. Genetic knockout of IER2 in the SW620 cell line was performed using CRISPR/Cas9 technology with guide RNAs (gRNA-1: TATCACTCCCGCATGCAGCG; gRNA-2: CGTCGTTTGCGGCTGACT; SW620/ko-IER2. Ko: knockout) following published protocols [12]. Control cell lines without guide RNA transfection (SW620/ko-ctrl. Ko-ctrl: knockout control) were also generated. Single-cell sorted clones were confirmed for alterations in the IER2-coding region by LGC Genomics GmbH (Berlin, Germany). Cell cultures were maintained at 37 °C with 5% CO_2_ and regularly tested for mycoplasma contamination using the MycoAlert Mycoplasma detection kit (Lonza, Basel, Switzerland). Cells were passaged every 3–4 days at approximately 80% confluence using Trypsin–EDTA (Thermo Fisher Scientific, Waltham, MA, USA), PBS, and fresh medium. G418 (Roche Applied Sciences, Basel, Switzerland) was used for continuous positive selection of MACC1-overexpressing cells. Cell counting was performed to maintain consistent flask density after cell seeding. Upon reaching 80% confluence, cells were detached from the flask using Trypsin–EDTA after washing with PBS. Trypsinization was stopped by adding media supplemented with 10% FBS. Cells were then washed off the vessel and mixed into a homogenous single-cell suspension. The trypan blue dye exclusion method was employed to assess cell viability, and the cell concentration was determined using an automated cell counter. Further dilutions were calculated based on the cell concentration and seeded accordingly.

### 2.2. RNA Extraction and RT-qPCR

For RT-qPCR quantifications, standard laboratory protocols were followed. Cells were plated at a density of 3 × 10^6^ cells per well in 6-well plates and grown until reaching 80% confluence. Total RNA was isolated using the Universal RNA Purification Kit (Roboklon, Berlin, Germany) according to the manufacturer’s instructions. Purified RNA (50 ng) was reverse-transcribed in a 20 μL reaction mixture containing 1.25 μM random hexamers, 1 × RT-buffer/dNTP mixture (1 mM each), 1 U/μL RNase inhibitor, and 10 U/μL MuMLV reverse transcriptase (all Biozym, Los Angeles, CA, USA). The reaction proceeded at 30 °C for 10 min, 50 °C for 40 min, and 99 °C for 5 min, followed by cooling at 4 °C (Biometra, Jena, Germany). The generated cDNA was subjected to gene-specific qPCR using the HotStart DNA Master SYBR Green I Kit (Biozym) following the manufacturer’s instructions. Gene-specific primer sets were used as follows: MACC1 fwd: 5′-TTC TTT TGA TTC CTC CGG TGA-3′; MACC1 rev: 5′-ACT CTG ATG GGC ATG TGC TG-3′; IER2 fwd: 5′-AGT GCA GAA AGA GGC ACA GC-3′; IER2 rev: 5′-ACC TTG GCC GAG AGG TAG AG-3′; RPII fwd: 5′-GAA GAT GGT GGG ATT TC-3′; RPII rev: 5′-GAA GGT GAA GGT CGG AGT-3′; G6PDH fwd: 5′-ATC GAC CAC TAC CTG GGC AA-3′; G6PDH rev: 5′-TTC TGC ATC ACG TCC CGG A-3′. Each PCR reaction was performed in a total volume of 10 μL in a LightCycler 480 system (Roche, Basel, Switzerland). Amplification consisted of an initial denaturation at 95 °C, followed by 40 cycles of denaturation (5 s; 95 °C) and primer annealing/elongation (45 s; 60 °C). Data analysis was conducted using LightCycler 480 Software release 1.5.0 SP3 (Roche Diagnostics, Basel, Switzerland). Mean values were calculated from duplicates, and the expression of each gene was normalized to the respective mean amount of glucoe-6-phosphate dehydrogenase (G6PDH) or human RNA polymerase II (RPII) (used as housekeeping genes).

### 2.3. Protein Extraction and Western Blot

A total of 5 × 10^6^ cells were cultured in 6-well plates and lysed with RIPA buffer on ice for 30 min after reaching the desired confluence. The protein concentration was determined using the Bicinchoninic Acid Protein Assay Reagent (Thermo Fisher Scientific) following the manufacturer’s instructions. Equal amounts of protein lysates were separated by SDS-PAGE and transferred onto PVDF membranes using a semi-dry blotting system (Biorad, Hercules, CA, USA). Membranes were blocked with 5% non-fat dry milk in TBS-T buffer for 1 h at room temperature. Subsequently, membranes were incubated overnight at 4 °C with primary antibodies against MACC1 (Sigma-Aldrich, Burlington, MA, USA, 1:1000 dilution, 5% BSA), IER2 (Sigma-Aldrich, 1:1000 dilution) or β-actin (Sigma-Aldrich, 1:10,000 dilution). After washing with TBS-T, membranes were incubated with HRP-conjugated secondary antibodies against rabbit IgG (Promega, Madison, WI, USA, 1:20,000 dilution) or mouse IgG (Thermo Fisher Scientific, 1:10,000 dilution) for 1 h at room temperature. Antibody–protein complexes were visualized using the WesternBright ECL substrate (Invitrogen, Carlsbad, CA, USA) and exposed to Fuji Medical X-Ray Film (Fujifilm, Tokyo, Japan). β-actin expression served as the loading control for protein quantification. All experiments were performed independently at least three times.

### 2.4. Co-Immunoprecipitation

For co-immunoprecipitation (Co-IP) assays, 5 × 10^6^ cells were seeded in 10 cm cell culture dishes and incubated for 24 h to form a cell monolayer. The monolayers were washed once with 1× PBS and then scraped off in 500 µL of ice-cold IP lysis buffer, transferred to sterile reaction tubes, and lysed for 30 min with intermittent vortexing at low intensity. Whole cell lysates containing proteins were obtained by centrifuging for 45 min at 14,000 rpm at 4 °C. The supernatants were collected and divided into 2 mg protein lysate aliquots. To precipitate the protein of interest, 2 µg of the respective target antibody was added to each lysate aliquot, which was then incubated overnight at 4 °C on a rotational shaker. The protein–antibody complexes were captured by adding 20 µL of G-agarose beads and incubating for 4 h at 4 °C on a rotational shaker. Afterwards, the G-agarose beads were precipitated by centrifugation at 500 rpm for 45 min at 4 °C. Supernatants were discarded, and the beads were washed five times with 200 µL of IP lysis buffer, followed by centrifugation at 2500 rpm for 5 min at 4 °C. After the final washing step, protein complexes were eluted with 30 µL of DTT-supplemented LDS buffer (1:10) at 99 °C for 10 min. Following another spin at 2500 rpm for 5 min at 4 °C, 20 µL of supernatant was subjected to Western blotting using the method described above.

### 2.5. Proliferation Assay

A total of 2 × 10^4^ cells were seeded in 96-well plates after passaging using the aforementioned technique, with each cell line plated in technical duplicates. The corresponding medium was added, and plates were placed in the IncuCyte^®^ ZOOM system. The incubator was maintained at a humidity of 5% CO_2_ and 37 °C. The system was calibrated to capture images every 2 h using the manufacturer’s software. After 96 h, the assay was terminated, and data points were collected, allowing for quantification of the cell confluence. The results were calculated from at least three independent experiments.

### 2.6. Survival Analysis and Data Mining of Expression Microarray Data

mRNA expressions of MACC1 and IER2 were measured using RT-qPCR using tumor samples of 60 colorectal cancer (CRC) patients at stages I, II, or III. Detailed information on patients and tumor tissue is provided in previous reports [11]. The Gene Expression Omnibus [13] (GEO) was used to search for publicly available expression data on CRC tumor microarrays. GDS4718 [14] was accessed, and expression data for target genes MACC1 and IER2 were obtained and normalized to expression data of glyceraldehyde 3-phosphate dehydrogenase (GAPDH). They were consecutively analyzed for direct or inverse correlation.

### 2.7. Statistical Analysis

Statistical analysis was performed using GraphPad Prism Version 6. Correlation analyses were performed using Pearson’s r. Comparison of the control with multiple groups was carried out using a one-way analysis of variance (ANOVA). Comparisons between two groups were carried out using the unpaired *t*-test. Survival rates were calculated with the Kaplan–Meier estimator. The cut-offs to distinguish low and high expression levels were determined using receiver–operator characteristic (ROC) analysis, which identified optimal cut-off values of 232.84 for MACC1 and 6.21 for IER2, maximizing the Youden Index. Statistical significance was set at *p* < 0.05 (*), *p* < 0.01 (**) and *p* < 0.001 (***).

## 3. Results

### 3.1. Correlation of MACC1 and IER2 Transcripts and Proteins In Silico

**Figure 1:**
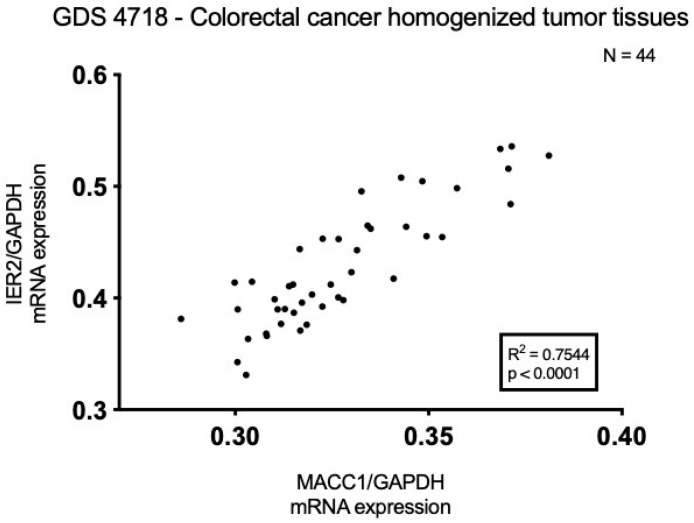
Correlation analysis for MACC1 and IER2 expressions in GEO dataset. Analysis using data from GEO dataset number GDS4718 [14] that positively correlates MACC1 and IER2 mRNA expressions. Normalization against GAPDH and mapped on XY plot. Significance was set at *p* < 0.05.

To determine the possible coordinate expressions of CRC biomarkers, we interrogated the NCBI’s Gene Expression Omnibus (GEO) database. GDS4718 [14] contains expression analysis from homogenized CRC tumors representing various disease stages with and without metastases from 44 patients. These analyses focused on MACC1 and IER2. The expression values of MACC1 and IER2 were extracted from the dataset, along with values for GAPDH, a stably expressed housekeeping gene. Normalized values were compiled and plotted accordingly, as shown in Figure 1. Using Pearson’s R correlation analysis, the IER2/MACC1 association revealed a significant positive correlation (R^2^ = 0.7544).

**Figure 2:**
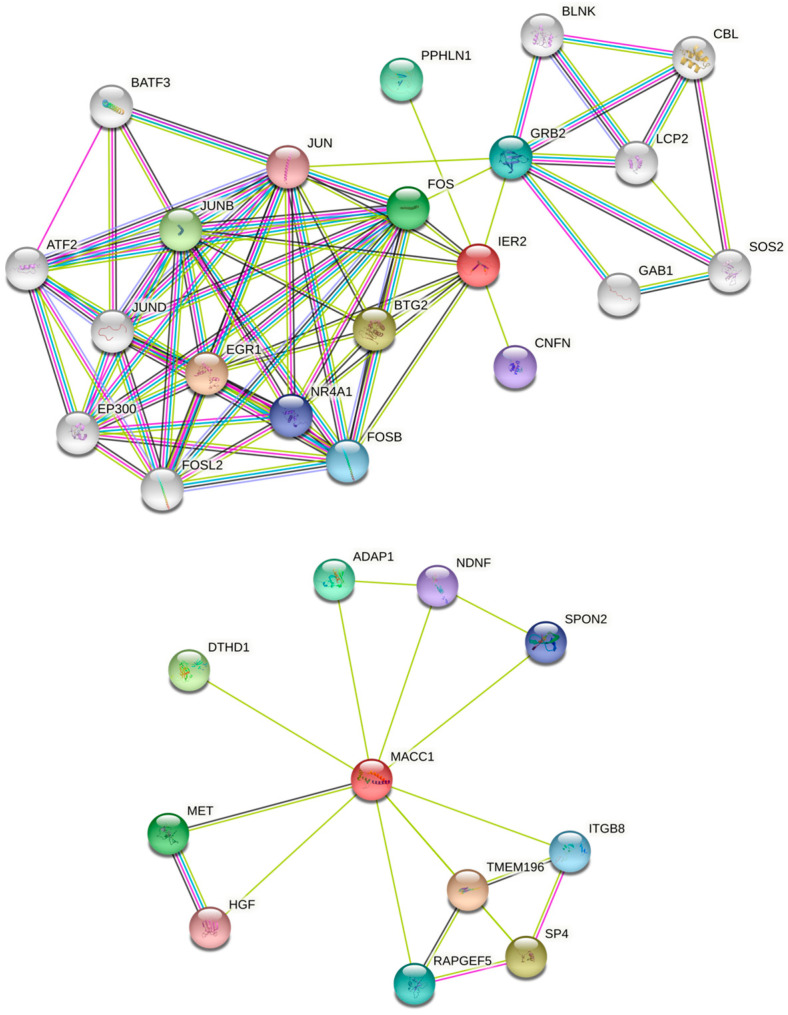
Representation of IER2 and MACC1 protein–protein association network. Predictions are from String Network [15,16] database. Highest predictive scores are for IER2 in text. (**Top**): IER2 String network. (**Bottom**): MACC1 String network. Lines represent protein–protein associations: light blue: known interactions from curated databases; pink: known interactions experimentally determined; green: predicted interactions based on gene neighborhood; red: predicted interactions based on gene fusions; royal blue: predicted interactions based on gene co-occurrence; light green: text mining; black: co-expression; lilac: protein homology.

To begin to understand the positive correlation between MACC1 and IER2 expressions, we conducted a further review of curated databases for predictive protein interactions and interaction sites. We consulted the STRING Network [15] (www.string-db.org) database. Searching for “IER2—homo sapiens” returned predicted functional partners such as EGR1, BTG2, JunB, FOS, and GRB2, among others (ordered from the highest to lowest assigned score: 0.850–0.636). The scores are not intended to indicate the strength of the interaction; rather, the confidence STRING determines whether the interactions are true based on available evidence. A value of 1 means the highest possible level of confidence. These associations were predicted through text mining and co-expression analysis, including anti-bait co-immunoprecipitation assays or tandem affinity purification assays, and therefore direct experimental validation is still required. A graphical representation of the IER2 and MACC1 networks according to the String database is shown in Figure 2. An important number of relevant cancer-related proteins are present in the interaction networks of both biomarkers, although no major overlaps between these networks was observed, initially predicting their roles in CRC progression to be individual.

### 3.2. MACC1 Regulates IER2 Expression in CRC Cells

**Figure 3:**
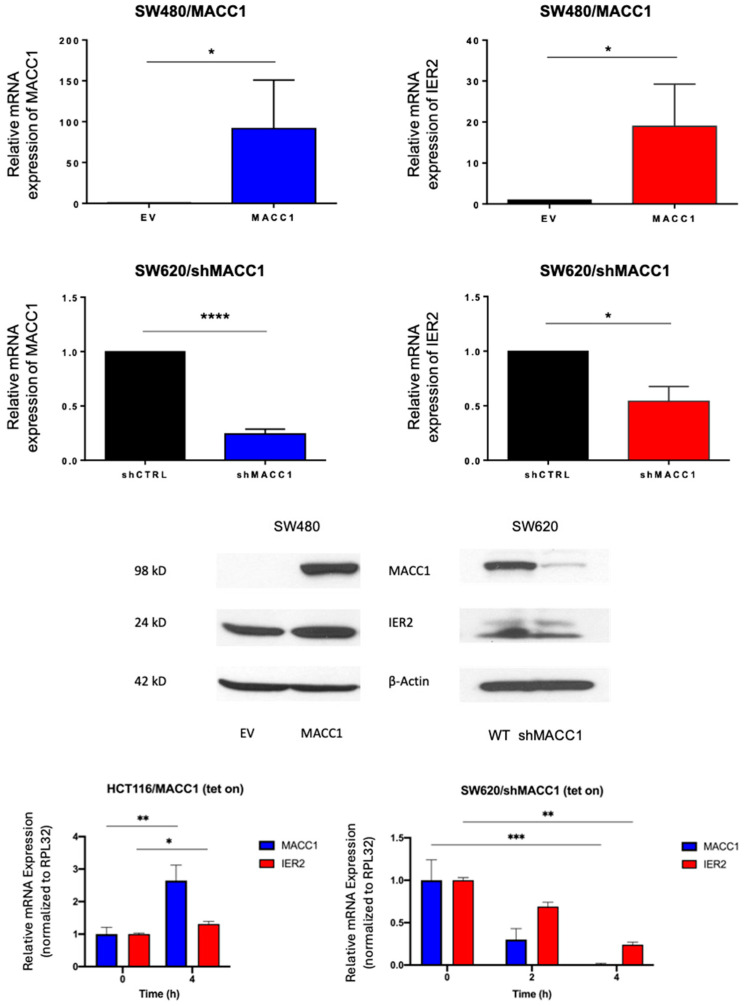
Co-expression of MACC1 and IER2 in colorectal cancer cells. (**Top**): Bar graphs showing mRNA expressions of MACC1 and IER2 in SW480/MACC1-transfected cells and SW480 EV control cells (**upper graphs**), as well as mRNA expressions of MACC1 and IER2 in SW620/shMACC1-transfected cells and SW620/shCtrl control cells (**lower graphs**). Significance was set at *p* < 0.05. (**Middle**): Western blots of SW480/EV and SW480/MACC1 cells (**left**), as well as of SW620/shMACC1 and SW620/shCtrl cells (**right**). MACC1 and IER2 were blotted in both cell lines. β-actin was used as loading control. (**Bottom**): Bar graphs showing mRNA expressions of MACC1 and IER2 after treatment with doxycycline in a tetracycline-induced HCT116/MACC1 system (**left**) and a tetracycline-induced SW620/shMACC1 system (**right**). Relative mRNA expressions have been normalized to housekeeping gene RPL32. Time is expressed in hours after exposure to the drug. Significance was set at *p* < 0.05. * *p* < 0.05, ** *p* < 0.01, *** *p* < 0.001, **** *p* < 0.00001. The original Western Bolt images can be found in the Supplementary Material.

To further understand the correlation between MACC1 and IER2 expressions in CRC patients, we investigated the impact of MACC1 on the expression of IER2. In previous work, we stably transfected MACC1 into human SW480 colon adenocarcinoma cells that lack endogenous MACC1 expression [11], resulting in a substantial increase in the MACC1 mRNA levels versus the empty vector (EV) control (*p* < 0.0286). Interestingly, a concurrent 20-fold upregulation of IER2 mRNA expression was also observed in the MACC1-expressing cells compared to the EV control (*p* < 0.0286), a finding corroborated at the protein level (Figure 3). Conversely, SW620 cells, derived from a metastasis of the same patient as the primary tumor of SW480, exhibit endogenously elevated MACC1 levels. Introduction of a MACC1-specific small hairpin RNA (shRNA) into the SW620 [11] cells led to a marked reduction in both MACC1 (76%, *p* < 0.0001) and IER2 (46%, *p* < 0.0278) mRNA expressions, which was also confirmed at the protein level (Figure 3).

To further substantiate the notion that MACC1 positively regulates IER2 expression, HCT116 cells harboring a tetracycline-inducible MACC1 promoter were utilized. The cells were induced with doxycycline, and the transcript levels were assessed via RT-qPCR at two time points (0 and 4 h) post-exposure. Parallel loss-of-function experiments were conducted using the SW620 cell line, using a short hairpin MACC1 (shMACC1) tetracycline-inducible construct, with an additional time point at 2 h.

Overexpression of MACC1 was achieved 4 h after doxycycline application, with a 2.65-fold increase in mRNA expression when compared to the basal expression before application of the antibiotic. Concurrently, IER2 exhibited a 30% increase in expression compared to the control. Conversely, in the MACC1 downregulation model, exposure to doxycycline for 2 h led to a significant 70% reduction in MACC1 mRNA expression compared to the control, with a concurrent non-significant 30% reduction trend in IER2 mRNA expression. Following 4 h of exposure, both MACC1 and IER2 mRNA expressions were significantly reduced by 99% and 76%, respectively (Figure 3).

### 3.3. IER2 Promotes Colony Formation and Sustains Proliferation in CRC Cells

**Figure 4:**
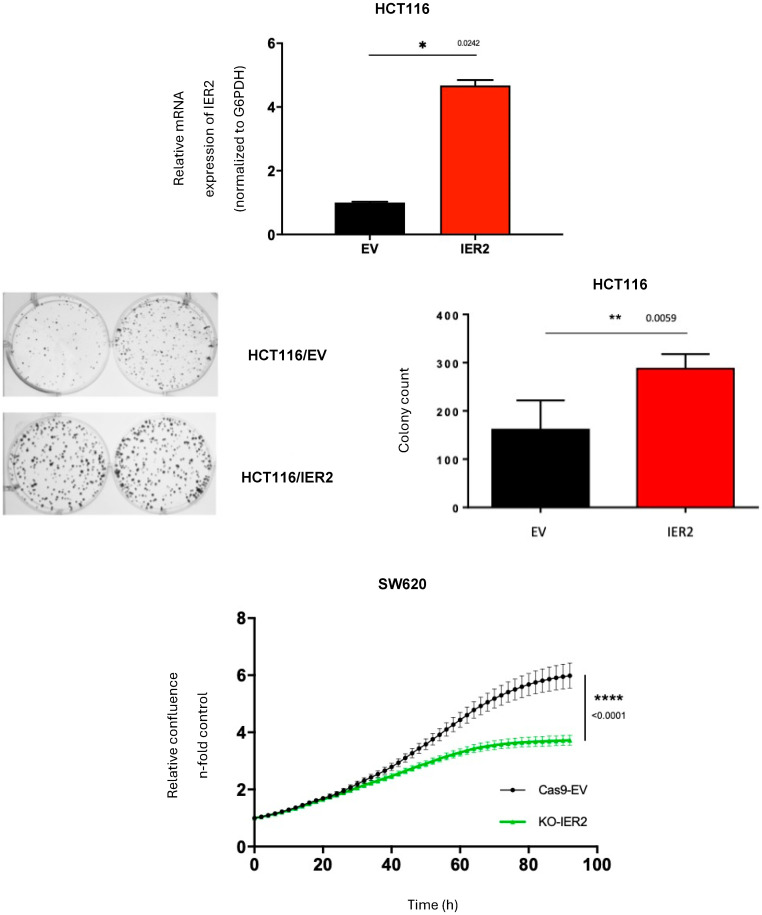
Promotion of colony formation and sustainment of proliferation in two CRC cell lines. (**Top**): Bar graph showing mRNA overexpression of IER2 in HCT116 cells transfected with IER2 (*p* < 0.0242). mRNA expression was normalized to housekeeping gene G6PDH. (**Middle**): Composition of six-well plate photographs to compare colony formation between IER2-overexpressing HCT116 (HCT116/IER2) cells and control (HCT116/ev) cells (**left**). Bar graph translating picture collage, where a significantly higher number of colonies were formed by IER2-overexpressing cells (*p* < 0.0059) (**right**). Significance was set at *p* < 0.05. (**Bottom**): Curvilinear graph depicting relative confluence against time in hours of SW620/Cas9-ev in comparison to SW620/KO-IER2. Proliferation was significantly decreased in absence of IER2. Time is indicated in hours. Results represent means + SEM of three independent experiments. Significant results were determined by two-way ANOVA and Sidak’s multiple comparison test. Significance was set at *p* < 0.05. * *p* < 0.05, ** *p* < 0.01, **** *p* < 0.00001.

To decipher the possible contribution of MACC1-induced IER2 expression to the tumorigenic properties of CRC cells, IER2 was ectopically expressed in HCT116 cells (HCT116/IER2 cell line) and colony formation assays were performed. As shown in Figure 4, a notable increase in the colony count was observed in cells ectopically expressing IER2 compared to those transfected with the empty vector control, indicating a significant enhancement in colony formation associated with IER2 overexpression. Consistent with these results, in loss-of-function experiments using IER2-depleted SW620 cells, a marked reduction in proliferation rates was observed in the IER2-depleted cells compared to the Cas9-EV control cells (Figure 4), indicating that IER2 expression is required for sustained proliferation.

### 3.4. IER2 and MACC1 Proteins Physically Interact

**Figure 5:**
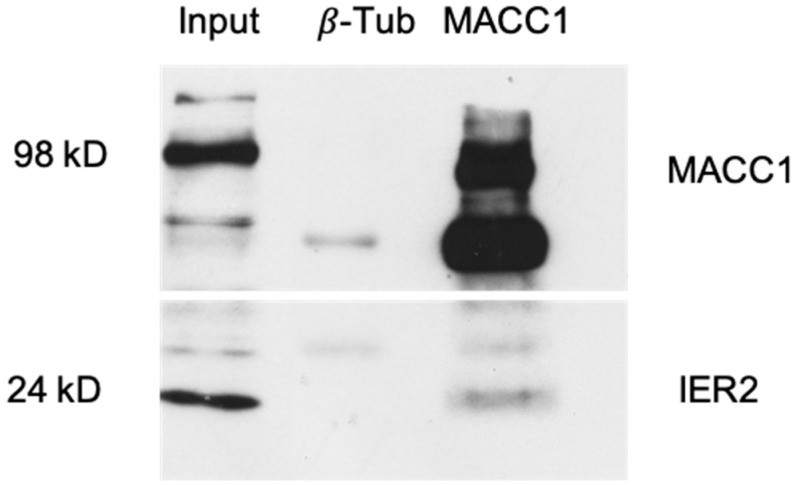
Co-immunoprecipitation assay using SW480/MACC1-transfected cells. Co-immunoprecipitation blot demonstrating successful IER2 pulldown and direct evidence for MACC1/IER2 protein interaction. Anti-β-tubulin was used as a negative control. IER2 molecular weight: 24 kDa; MACC1 molecular weight: 98 kDa. The original Western Bolt images can be found in the Supplementary Material.

Given the functional contributions of the IER2 and MACC1 proteins to the promotion of the migratory and invasive behavior of cancer cells, as well as their complementary interaction networks (Figure 2), we next conducted co-immunoprecipitation assays to test whether MACC1 and IER2 physically interact. To this end, MACC1 was immunoprecipitated from lysates of SW480/MACC1-transfected cells, and the immunoprecipitated material was assessed by Western blot for the presence of IER2. Immunoprecipitation with anti-β-tubulin antibodies served as a negative control. As shown in Figure 5, IER2 was present in immunoprecipitants using the MACC1 antibody but not in the anti-β-tubulin antibody control, indicating an interaction between the MACC1 and IER2 proteins. The data provide the first evidence for a molecular link between immediate early transcriptional responses and sustained pro-metastatic signaling.

### 3.5. Coordinate Expressions of MACC1 and IER2 Correlate with Poorest Prognosis in CRC Patients

**Figure 6:**
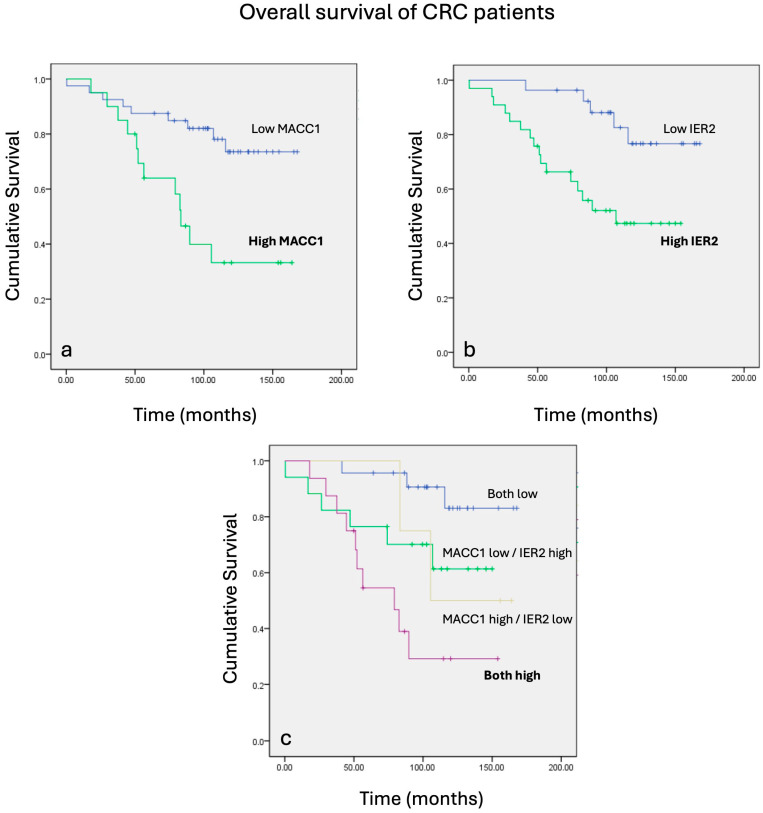
Kaplan–Meier plots showing MACC1 and IER2 mRNA expressions against cumulative survival. Patients were ranked according to high or low mRNA expressions of IER2 and MACC1 with ROC. (**a**) Higher IER2 mRNA expression leads to worst cumulative survival. (**b**) Higher MACC1 mRNA expression translates into poorer cumulative survival. (**c**) Patients who expressed high levels of both MACC1 and IER2 were also patients with worst outcomes measured in cumulative survival over months.

Building on the pivotal role of IER2 and MACC1 in promoting the progression and metastasis of CRC, we next determined the impact of MACC1 and IER2 expressions individually and together for patient prognosis. To this end, the transcript levels of these genes in samples from 60 CRC patients were investigated, and Kaplan–Meier plots were utilized to analyze the cumulative survival against time in months. Patients with higher IER2 expression had significantly shorter survival compared to those with low IER2 expression. High tumoral expression of IER2 mRNA was associated with a 10-year survival rate of approximately 50%, while patients with low IER2 expression showed a 10-year survival rate of approximately 80% (Figure 6). Similarly, high MACC1 expression values were associated with shorter survival for these patients. High tumoral expression of MACC1 resulted in a 10-year OS of around 30%, whereas patients with low MACC1 expression had a 10-year overall survival rate close to 70% (Figure 6). Interestingly, patients with high expressions of both MACC1 and IER2 exhibited significantly shorter cumulative survival, whereas those with low concentrations of both markers showed the longest survival: a 5-year OS of 50% and a 10-year OS of 30% was observed for patients with both high IER2 and MACC1 mRNA expression levels. Conversely, when the expressions of both genes were low, the 5- and 10-year OS rates were over 90% (Figure 6).

## 4. Discussion

The study of the genetic and epigenetic alterations that underlie CRC have given clinicians a wide range of predictive molecular markers, several of which are now used in the routine care of CRC patients [17]. Understanding the interactions between these biomarkers is fundamentally reshaping our understanding of CRC biology and treatment. Rather than viewing biomarkers as independent entities, network-based approaches are revealing how molecular relationships drive disease progression, treatment resistance, and patient outcomes [18]. These developments have a number of important consequences for advancing precision medicine. For example, prognostic accuracy will improve when biomarker interactions are considered. Treatment strategies must consider biomarker networks rather than individual markers and should target network vulnerabilities rather than single pathways. As resistance mechanisms often involve pathway crosstalk, combination approaches based on an understanding of biomarker interactions will also help to combat therapy failure. The functional interplay between the CRC biomarkers MACC1 and IER2 that we report here contributes to our understanding of CRC biomarker networks and has the potential to contribute to improved prognostic accuracy and treatment strategies.

With regard to prognosis, expression of either MACC1 or IER2 has previously been shown to correlate with poor survival in CRC patients. For example, MACC1 mRNA expression was found to be higher in CRC tumors that later metastasized (metachronously) compared to those that did not [11], and IER2 overexpression correlated with poor metastasis-free and overall survival in patients with CRC [7]. Here, we confirm and extend these findings. Importantly, we show that the concomitant expressions of both MACC1 and IER2 are a stronger predictor of poor prognosis than the expression of either biomarker individually. These clinical data provide support for the notion that MACC1 and IER2 act in a complementary way to promote CRC progression and metastasis. They also indicate that assessing the expressions of both biomarkers should provide superior diagnostic accuracy compared to assessing single biomarker expression. Additionally, they suggest that the therapeutic targeting of MACC1 and IER2 concomitantly may be more effective than targeting only one of these biomarkers.

In silico analysis utilizing the GEO database revealed a robust positive correlation between MACC1 mRNA expression and IER2 mRNA expression. Consistently, in loss- and gain-of-function experiments, we found evidence that MACC1 expression upregulates IER2 transcription and protein expression. MACC1 is known to regulate the transcription of a number of target genes by either regulating the activity of signaling pathways [4] or by acting as a transcriptional regulator through binding directly to the promoter of target genes such as MET [11] and S100P [6]. Future work will focus on investigating the precise mechanism(s) through which MACC1 stimulates the expression of IER2.

IER2 is described as one of several genes cited as immediate early genes (IEG), which have the biological privilege of being rapidly induced within minutes after the chemical and/or physical stimulation of cells, even in the presence of protein synthesis inhibitors [19]. Other notable IEGs are the AP-1 components Fos and Jun, Krüppel-like factors, zink-finger proteins, and others. A study in hematopoietic stem cells proved Fos to be a direct promoter of IER2 expression upon proliferative stress [19].

In tumor tissue, IER2 demarcates activated fibroblasts (i.e., proliferating, migrating cancer-associated fibroblasts—CAF) and cancer cells, as shown in CRC and melanoma [7,10]. Hypoxia is a robust inducer of IER2 expression in CAFs of recurring chordomas [20]. MACC1 is a causal biomarker of hypoxia in tumors and improves survival of cancer cells under hypoxic conditions through the Warburg effect: by shifting Glucose Transporter Typ 1 (GLUT1) transmembrane proteins to the cell membrane, MACC1 improves the glucose uptake of cancer cells [21,22]. This is in line with the finding that MACC1 overexpression enables cancer cells to endure the hypoxigenic processes of proliferative migration and invasion [6].

Increased expression of IER2 by MACC1 will likely contribute to the promotion of CRC progression, as we found that IER2 expression stimulates both colony formation and sustained proliferation by CRC cells. IER2 is recognized as a regulator of the G1-S cell cycle transition. A recent Bayesian model, employed for predicting cell cycle components, successfully identified known factors such as CDC25A and highlighted the involvement of IER2 in the G1-S transition [23]. Consistent with this, studies have demonstrated an interaction between IER2 and CDC25A facilitated by the 14-3-3 protein (a regulatory protein) [24]. This interaction occurs at the T507 residue within CDC25A, situated within a cyclin B1/cyclin-dependent kinase 1 (CDK1) site in the C terminus of CDC25A [24]. Through dephosphorylation of T507, IER2 disrupts the association of CDC25A with 14-3-3, thereby activating CDC25A. CDC25A, essential for cell proliferation, is tightly regulated, and its dysregulation can significantly impact the cell cycle by promoting the dephosphorylation and activation of CDKs required for the G1-S transition and G2-M checkpoint [25]. Hence, aberrant cell cycle regulation mediated by CDC25A dysfunction may underlie the enhanced colony-forming ability observed in cells overexpressing IER2. Furthermore, the IER2-induced hyperphosphorylation of the downstream mTOR effector S6 kinase (S6k) [8] has been implicated in the regulation of cellular proliferation [26], and thus reduced S6k phosphorylation may also contribute to the reduction in proliferation observed after knockdown of IER2.

Although mechanistically we show here that MACC1 expression promotes IER2 expression, which, in turn, fosters colony formation and cell proliferation, the clinical data indicate that the expressions of both IER2 and MACC1 have a more pronounced prognostic significance than either biomarker alone. The latter observation suggests that IER2 and MACC1 have complementary effects on poor patient prognosis. Our STRING network analysis provides some initial support for this notion. However, it is notable that the STRING analysis did not flag up a number of proteins that have been shown to physically interact with either MACC1 or IER2. MACC1, for example, physically interacts with a number of intracellular kinases, most notably MEK1 [27], clathrin-mediated endocytosis proteins that regulate receptor trafficking [28], as well as transcriptional regulators and signal transduction proteins, such as SNAI1 and YWHAE [29]. The MACC1 SH3 domain and proline-rich regions play a key role in these interactions. IER2 physically interacts with the protein phosphatase PP2A, the cell cycle regulator CDC25A, the tumor suppressor protein RB, and the FGF-binding protein FIBP1 [30,31]. The finding that the MACC1 and IER2 proteins themselves physically interact may therefore also be relevant in the context of the complementary activities of these biomarkers, as the interaction would be expected to act as a hub, bringing together the different proteins with which the two biomarkers interact. In this regard, it is interesting to note that MACC1 directly interacts with 14-3-3 epsilon (YWHAE), which activates the PI3K/Akt pathway [4]. IER2 also activates the PI3K/AKT pathway via modulation of the AKT through PP2A [8]. Furthermore, IER2 dephosphorylates its interaction partner CDC25A, leading to dissociation of CDC25A from 14-3-3 and, subsequently, increased CDC25A activity on CDK1/cyclin B1 and cell cycle progression [31]. PP2A critically affects signaling pathways that regulate mitogen activated protein kinase (MAPK) and nuclear factor kappa B (NF-κB) [32,33], which, in turn, lead to transcriptional control of MACC1.

We speculate that IER2 is likely to stimulate MACC1 expression via AP-1. Our group established that AP-1, a heterodimer of c-jun and c-fos, binds to the promoter and induces transcription of MACC1 [6]. IER2 enhances adhesiveness of hepatocellular cancer (HCC) cells through promotion of integrin-β 1 (ITGB1) expression [9]. During outside-in signaling (i.e., upon ECM binding), ITGB1 broadly activates AP-1/c-Jun signaling via both FAK-Src-MEK/ERK as well as PI3K-Akt-NF-κB [34]. NF-κB induces MACC1 downstream of TNF-α signaling and via the AP-1 component c-Jun [35]. IER2 has been independently shown to induce senescence via MEK/ERK- and Akt signaling [10]. A possible alternative effect to senescence is the induction of motility by IER2, demonstrated in NIH 3T3 fibroblasts. Motile cells experience an increase in ECM contact, which intensifies integrin-dependent outside-in signaling via MEK and Akt [7]. These findings make IER2 likely to induce transcription of MACC1 via AP-1, particularly in cells at the invasive tumor front. These observations serve to illustrate how an interaction between MACC1 and IER2 could synergistically promote CRC progression. Nevertheless, additional work is required to substantiate and understand the significance of the physical interaction between the both of them.

Finally, this work is not without its limitations. These hypotheses have not been tested with animal models, and the clinical sample size for the in silico models used was limited. We postulate de novo mechanistic functions of an IER2/MACC1 axis that still needs further validation. Studies looking into the interaction of both proteins via SH3 domains could be informative. Regretfully, the IER2 promoter has not yet been identified, hindering successful chromatin immunoprecipitation experiments. Furthermore, functional assays with manipulation of both genes could elucidate a direct mechanistic relationship between both biomarkers and provide insights into future therapeutic strategies targeting the MACC1/IER2 interface.

## 5. Conclusions

In summary, our study provides insights into regulatory interactions between MACC1 and IER2, two functionally relevant biomarkers of CRC. Mechanistically, these interactions are expected to allow MACC1 and IER2 to act synergistically to promote tumor progression, a notion which is supported by the observation that the concomitant expressions of both biomarkers predict the poorest survival of CRC patients. Further studies on this axis should prove beneficial in future translational applications in patient diagnosis, prognostics or treatment.

## Data Availability

The original contributions presented in this study are included in the article. Further inquiries can be directed to the corresponding author.

## Supplementary Materials

The following supporting information can be downloaded at: https://www.mdpi.com/article/10.3390/biom16030398/s1, The original western blot figure.
