# Supplementary figures and images for: The Regulatory Interplay of the Colorectal Cancer Biomarkers MACC1 and IER2 and Its Impact on Metastatic Cancer Survival
Source: Biomolecules. 2026 Mar 7;16(3):398. doi: 10.3390/biom16030398 (PMC13024734; doi:10.3390/biom16030398)

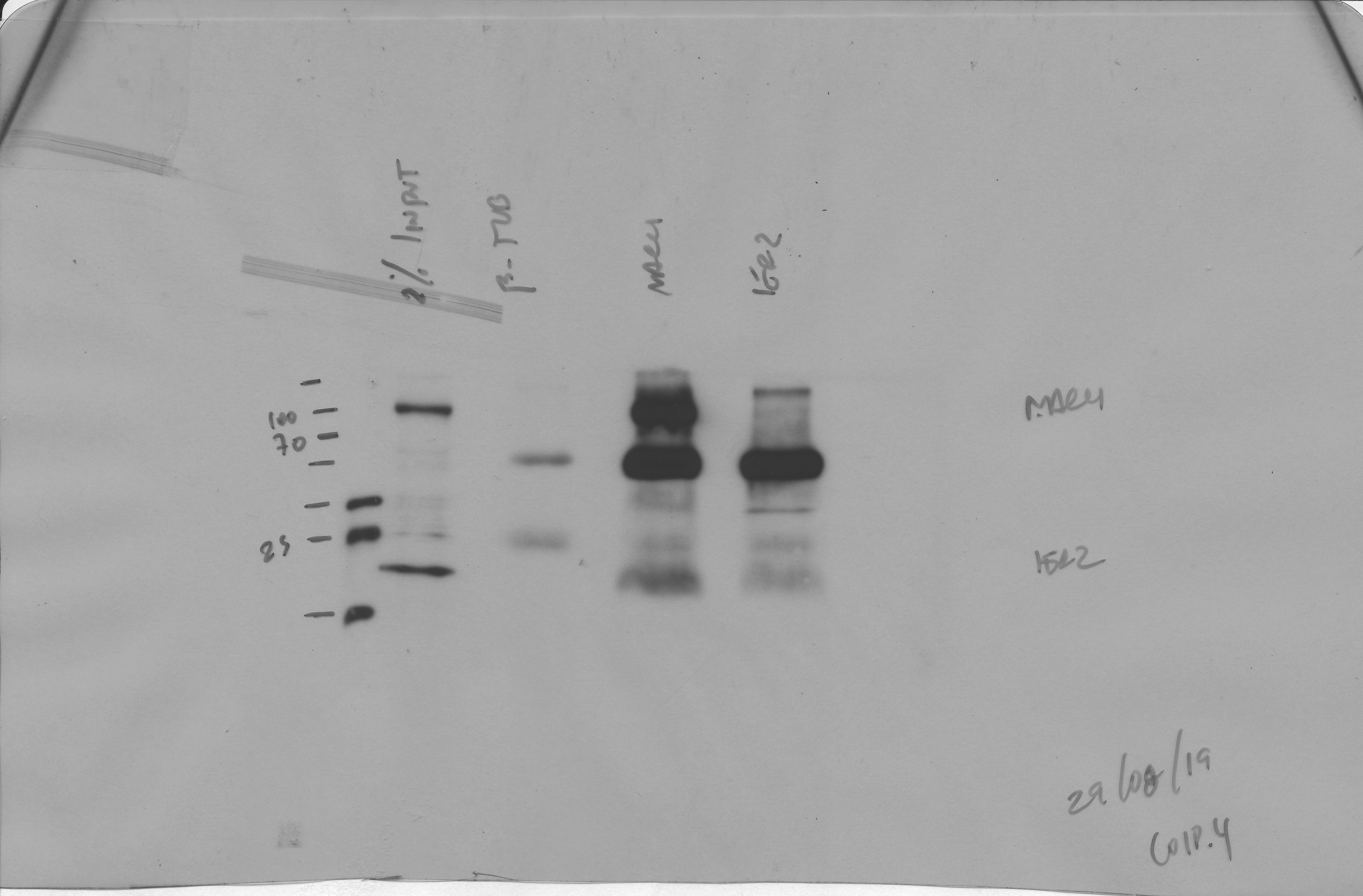

Supplement: Supplementary file 1 [file biomolecules-16-00398-s001.zip › CoIP-4_29082019_0001.jpg]

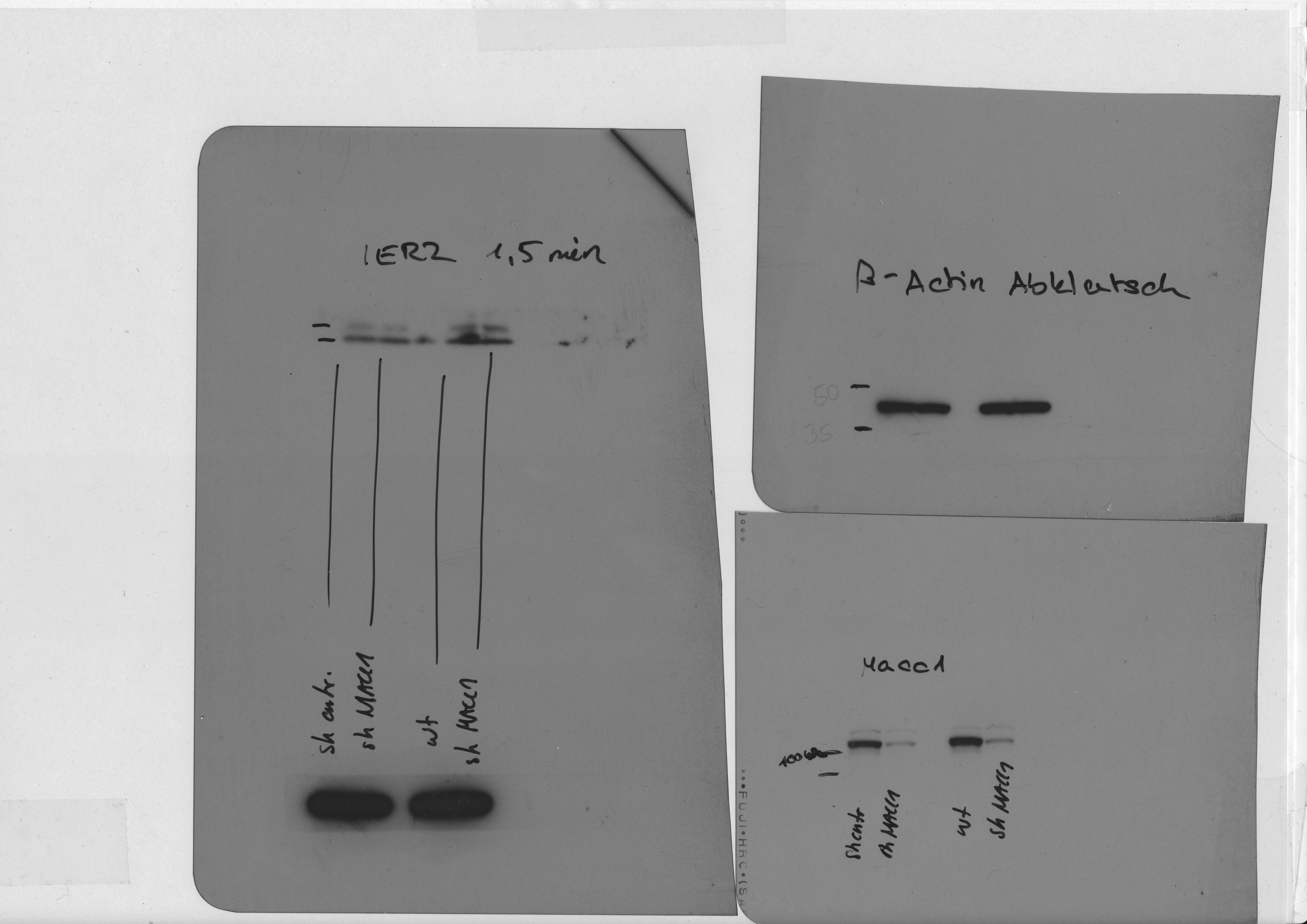

Supplement: Supplementary file 1 [file biomolecules-16-00398-s001.zip › SW620 sh klone MACC1 IER2 B-Actin[2] copy.jpg]

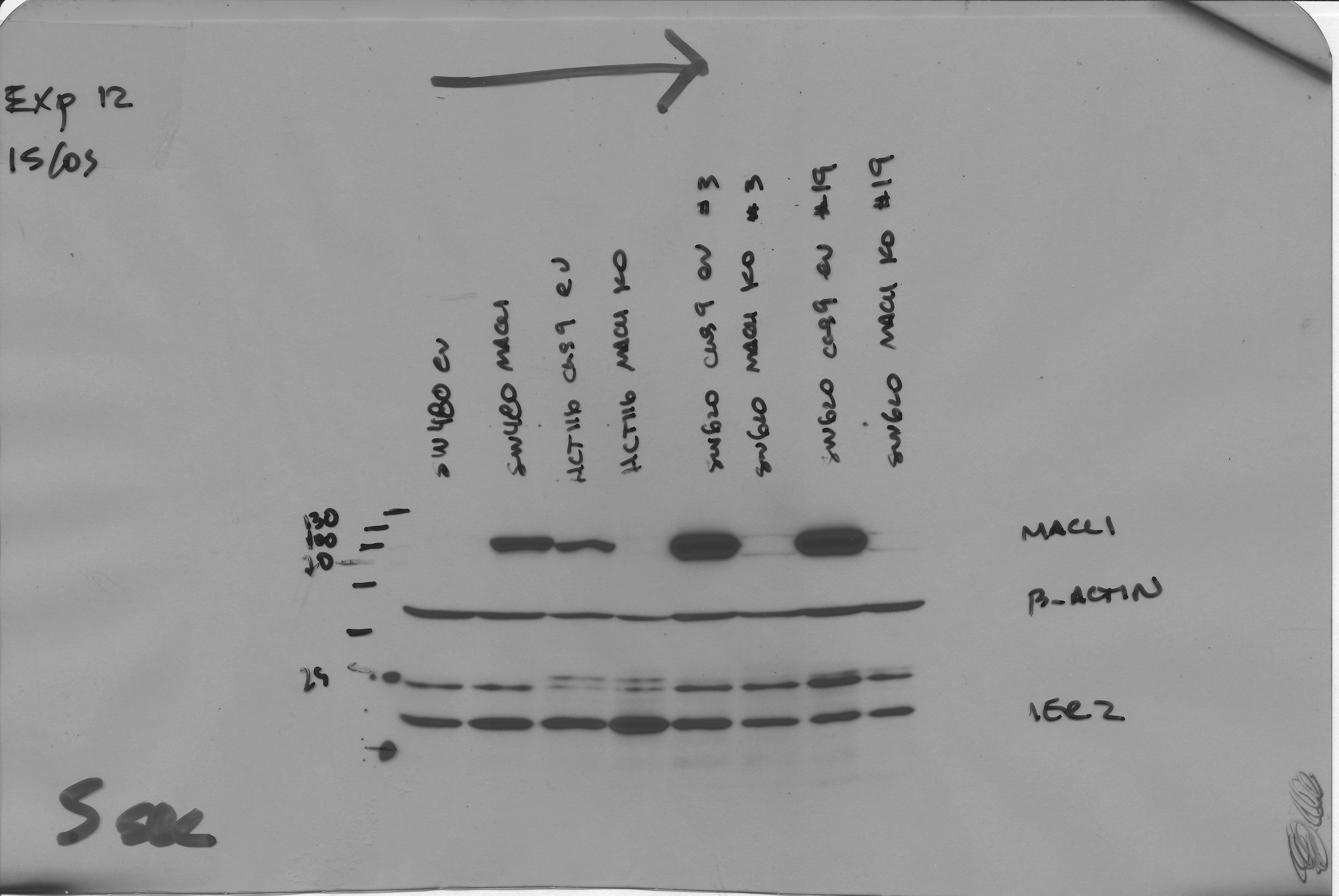

Supplement: Supplementary file 1 [file biomolecules-16-00398-s001.zip › Exp 12 SW480-HCT116-SW620_0001.jpg]
